# Computational analysis of variation in *C. elegans ugts*

**DOI:** 10.17912/micropub.biology.000819

**Published:** 2023-08-07

**Authors:** Muhammad Zaka Asif, Maci C. Benveniste, Kyra D. Chism, Ari L. Levin, Deanna Lanier, Rockford E. Watkins, Rahil Taujale, Niyelle Tucker, Arthur S. Edison

**Affiliations:** 1 Department of Biochemistry & Molecular Biology, University of Georgia, Athens, Georgia, United States; 2 Complex Carbohydrate Research Center, University of Georgia, Athens, Georgia, United States; 3 Department of Genetics, University of Georgia, Athens, Georgia, United States; 4 Institute of Bioinformatics, University of Georgia, Athens, Georgia, United States

## Abstract

*Caenorhabditis elegans* are free-living nematodes with a relatively short life cycle and a wealth of genomic information across multiple databases. Uridine diphosphate-glycosyltransferases (UGTs) are a family of enzymes involved in Phase II modification of xenobiotics in *C. elegans* , which is the addition of a sizeable water-soluble molecule to a xenobiotic to allow for its excretion out of a cell. Little is known about the variation in UGTs across wild isolates and how that might affect their innate immune response. We analyzed the diversity in *ugt* genes across *C. elegans* isolates from different geographical locations from the *Caenorhabditis elegans* Natural Diversity Resource (CaeNDR) database. This was accomplished using whole genome data and data identifying genome regions as hyper-divergent for each isotype. We implemented three steps to identify *ugt* genes and make inferences based on their variation. First, we created a catalog of UGTs in the N2 reference strain and used them to create a phylogenetic tree that depicts the relationships between the UGT protein sequences. We then quantified *ugt* variation using the strains from the CaeNDR database and used their data to remove hyper-divergent *ugt* genes. The third step was to catalog the occurrence of minor allele frequency (MAF) > 0.05 for all the *ugts* to compare how that aligned with genes classified as hyper-divergent by CaeNDR. Of the 67 *ugt* genes analyzed, 18 were hyper-divergent. This research will help improve our understanding of *ugt* variation in *C. elegans* .

**Figure 1. ugt variation in C. elegans:**
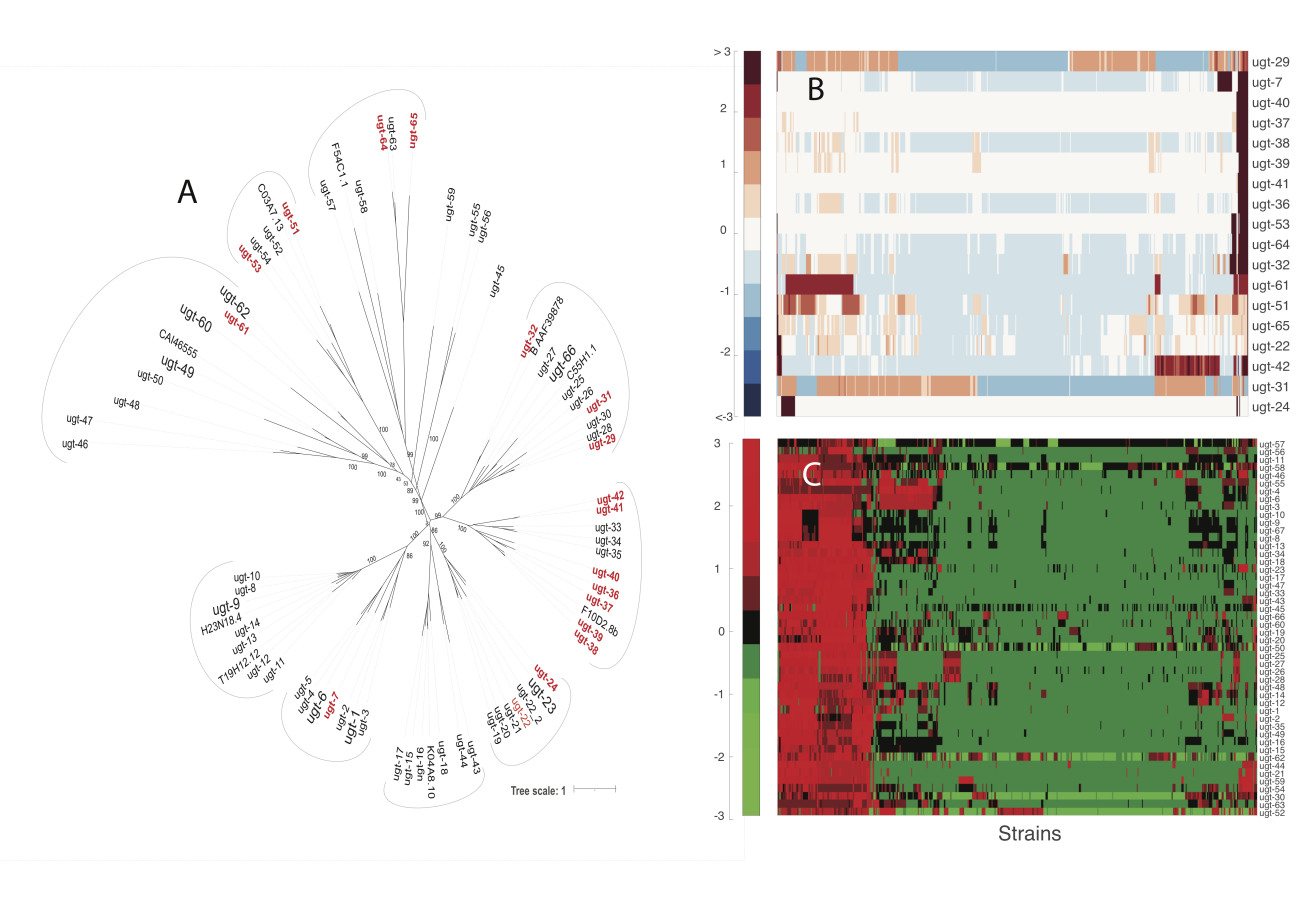
**A** : Phylogenetic tree of the known UGTs in *C. elegans* . The hyper-divergent UGTs are enlarged and highlighted in red. **B** : Hierarchical cluster of the known hyper-divergent *ugt* genes. A heatmap of the z-score of the variation is plotted. **C** : Hierarchical cluster of the non-hyper-divergent *ugt* genes. A heatmap of the z-score of the variation is plotted.

## Description

*C. elegans* has about 250 glycosyltransferases (Kellokumpu, Hassinen, & Glumoff, 2016) , and the *ugt* family of 67 genes are responsible for the glycosylation of small molecule xenobiotics (Asif et al., In Preparation; Hartman et al., 2021; Laing et al., 2010; Liu, Samuel, Breen, & Ruvkun, 2014) . We quantified the variation in 67 *ugt* genes across *C. elegans* isotypes using N2 as the reference strain (Cook, Zdraljevic, Roberts, & Andersen, 2017) . Regions with higher-than-average concentrated genomic variation than N2 are hyper-divergent (Lee et al., 2021) . According to Lee et al., hyper-divergent regions had nine consecutive bins of over 1kb equal to 16 single nucleotide variants (SNVs)/indels or lower than 35% read depth to the genome-wide average (Lee et al., 2021) . These hyper-divergent regions in their respective UGTs were identified and removed from our analysis.

We used the Multiple Alignment using Fast Fourier Transform **(** MAFFT) tool first to align the amino acid sequences into a multiple sequence alignment which was then used to generate a phylogenetic tree via the iqtree tool (Fig.1A) (Nguyen, Schmidt, von Haeseler, & Minh, 2015) . This phylogenetic tree groups evolutionarily related UGTs into clades that can be used to infer functional similarities. Ten clades were identified, providing an evolution-evidenced grouping of functionally related UGTs. Seven of the ten clades had at least one hyper-divergent UGT (shown in red in Figure 1A ), and most were grouped into a single clade.

Figure 1B shows hyper -divergent genes that were removed from our analysis. We identified hyper-divergent genomic regions from Lee et al., which were defined as hyper-divergent regions in more than 5% of all isotypes. From that, we identified *ugt* s that lay within these regions. Those *ugt* s were defined as hyper-divergent. We verified hyper-divergent genes by looking at the minor allele frequency (MAF) > 0.05 for SNVs and found that the genes with the highest numbers of SNVs with MAF > 0.05 tended to be hyper-divergent. It is important to note that genes not classified as hyper-divergent by Lee et al. still have some isotypes with MAF > 0.05 bases.

Using the cluster gram function in MATLAB™, we visualized the number of mutations in each hyper-divergent *ugt* . Z-score normalization was performed on the *ugts,* and a Euclidean distance metric was employed to measure similarity or dissimilarity based on the magnitude of differences between *ugts* and isotypes. The resulting standardized and clustered data were represented as a heatmap in Figure 1B . This heatmap’s dark colors represent values greater than three and lower than -3.

Figure 1C is the cluster gram for the non -hyper-divergent genes, as described above. The red-green colormap was chosen to contrast with Figure 1B of the hyper -divergent regions. The color bar indicates the z-score of the variation for the *ugts* . The non-hyper-divergent genes had a lower number of mutations than the hyper-divergent genes. The gene with the highest number of mutations was *ugt-12 .* Furthermore, the isotypes with the highest frequency of mutations (shown in the red region to the left in Figure 1C ) mostly are from Hawaii, indicating that isolation from other *C. elegans* isotypes allows for more divergent evolution.

Non-hyper-divergent *ugts* are an area of interest for future studies. Given the quantified genomic variation across isotypes from many locations, our results suggest that multiple environmental factors, such as climate, bacteria, pathogens, and environmental toxins, affect the variation.

## Methods

**Generating the Phylogenetic Tree** : We collected 77 UGT amino acid sequences from the publicly available CAZy and Wormbase databases. Next, we used Multiple Alignment using Fast Fourier Transform (MAFFT Alignment) tool to align the amino acid sequences for the UGTs. Then, we generated the phylogenetic tree using the iqtree tool. We visualized the phylogenetic tree using the online tool called the Interactive Tree of Life (iTOL) ( Fig. 1A ) (Nguyen et al., 2015) .

**Identifying Genomic Variation of CaeNDR Strains Compared to N2** : Using the information gathered above, we generated a Python script in Jupyter Notebook™ to parse CaeNDR's hard-filtered variants vcf file ***WI.20220216.impute.isotype.vcf.gz*** (released 20200815) and extracted the number of variants and location of mutations in *ugt* regions for 550 isotypes compared to the N2 reference genome. The genomes of the isotypes were aligned and compared to the N2 genome.

**Removal of Hyperdivergent Regions from Analysis** : Using the CaeNDR hyper-divergent region data file (20220216.bed), we created a Python script using Jupyter Notebook™ to determine which *ugt* s had hyper-divergent isotypes. Our data included regions that partially fell in a hyper-divergent range or had complete overlap. A table was created with our data. We further separated it into two Excel files containing non-hyper-divergent and hyper-divergent strains and UGTs with the number of base pair mutations across isotypes. If a gene from an isotype partially or fully fell into a hyper-divergent region, it was considered hyper-divergent for analysis purposes. All others were considered non-hyper-divergent.

**Creation of Heatmap** : Once the hyper-divergent regions were identified, a spreadsheet was created for both hyper-divergent and non-hyper-divergent genes. Both files contained the *ugt* names on the rows and the strain names in the columns. The total number of nucleotide variations in each strain for each *ugt* was also added. The non-hyper-divergent and hyper-divergent spreadsheet files were added to a working MATLAB® script. They were then used to create two cluster grams to help visualize variation trends, if any. ( Fig. 1B and Fig . 1C). Figure 1B was given a red -blue color map, whereas 1C was given a red-green to help differentiate between the data. All scripts are available on GitHub.
